# Fatal overdose prevention and experience with naloxone: A cross-sectional study from a community-based cohort of people who inject drugs in Baltimore, Maryland

**DOI:** 10.1371/journal.pone.0230127

**Published:** 2020-03-11

**Authors:** Megan Buresh, Rachel E. Gicquelais, Jacquie Astemborski, Gregory D. Kirk, Shruti H. Mehta, Becky L. Genberg

**Affiliations:** 1 Division of Addiction Medicine, Johns Hopkins School of Medicine, Baltimore, Maryland, United States of America; 2 Department of Epidemiology, Johns Hopkins Bloomberg School of Public Health, Baltimore, Maryland, United States of America; 3 Division of Infectious Diseases, Johns Hopkins University School of Medicine, Baltimore, Maryland, United States of America; University of California San Diego School of Medicine, UNITED STATES

## Abstract

**Introduction:**

Overdose is a leading cause of death in the United States, especially among people who inject drugs (PWID). Improving naloxone access and carrying among PWID may offset recent increases in overdose mortality associated with the influx of synthetic opioids in the drug market. This study characterized prevalence and correlates of several naloxone outcomes among PWID.

**Methods:**

During 2018, a survey to assess experience with naloxone was administered to 915 participants in the AIDS Linked to the IntraVenous Experience (ALIVE) study, an ongoing community-based observational cohort of people who currently inject or formerly injected drugs in Baltimore, Maryland. We examined the associations of naloxone outcomes (training, supply, use, and regular possession) with socio-demographic, substance use and healthcare utilization factors among PWID in order to characterize gaps in naloxone implementation among this high-risk population.

**Results:**

Median age was 56 years, 34% were female, 85% were African American, and 31% recently injected. In the past six months, 46% (n = 421) reported receiving training in overdose prevention, 38% (n = 346) had received a supply of naloxone, 9% (n = 85) had administered naloxone, and 9% (n = 82) reported usually carrying a supply of naloxone. Recent non-fatal overdose was not associated with any naloxone outcomes in adjusted analysis. Active opioid use (aOR = 2.10, 95% CI: 1.03, 4.28) and recent treatment of alcohol or substance use disorder (aOR = 2.01, 95% CI: 1.13, 3.56) were associated with regularly carrying naloxone.

**Conclusion:**

Further work is needed to encourage PWID to carry and effectively use naloxone to decrease rates of fatal opioid overdose. While accessing treatment for substance use disorder was positively associated with carrying naloxone, EMS response to 911 calls for overdose, the emergency department, and syringe services programs may be settings in which naloxone access and carrying could be encouraged among PWID.

## Introduction

Overdose is a leading cause of morbidity and mortality among people who inject drugs (PWID) [1] and opioid-related mortality rates have continued to increase among all age groups in the US [2]. Given the 71% yearly increase in overdose mortality between 2013 and 2017 in the US, attributed largely to synthetic opioids other than methadone, there is an urgent need to strengthen and expand overdose prevention programs. Naloxone, the opioid antagonist that reverses opioid overdoses, is a key tool in curbing opioid overdose mortality. Naloxone has been used by the medical community since the early 1970s and was first distributed to high-risk individuals in the late 1990s. From 1996 to June 2014, 136 opioid overdose education and naloxone distribution (OEND) programs in the US have provided naloxone kits to 152,283 people and reported 26,463 potential opioid overdose reversals [3].

As OEND programs continue to expand, research has consistently demonstrated their safety and effectiveness [4–11]. Most importantly, OEND participation is associated with improved ability of participants to respond appropriately to overdose and safely administer naloxone in order to prevent fatal overdoses [12]. Participants of OEND have been characterized as predominantly homeless PWID, who have commonly received drug treatment and witnessed overdoses [13–17], suggesting that training efforts may be adequately reaching vulnerable populations. In fact, in a recent study among persons who currently use or formerly used heroin in Baltimore in 2018, 90% had heard of naloxone and the majority had received naloxone training, but only one-third of those aware of naloxone reported regularly carrying it [18]. This suggests that despite good coverage of educational campaigns, additional work may be needed to ensure adequate supply and to encourage those most likely to experience an overdose to habitually carry naloxone. Additional research to understand characteristics associated with gaps in naloxone access can inform targeted interventions to those most likely to respond.

Many states, including Maryland, have attempted to further expand naloxone availability via third party prescribing and standing order laws, and have encouraged naloxone use through Good Samaritan Laws, which legally protect persons who respond to an overdose from criminal charges. Third party prescribing and standing order laws tend to be in jurisdictions with more extensive OEND implementation [19] and states with naloxone laws including Good Samaritan laws have experienced declines in opioid overdose mortality [20]. Maryland’s Good Samaritan law was enacted in 2015 and an unrestricted naloxone standing order followed in 2017 (though naloxone has been available to those who completed a training since 2015). The Baltimore City Health Department (BCHD) launched Staying Alive, an OEND program, in April 2004, with the goal of training PWID, drug treatment clients and providers, inmates and corrections officers. BCHD has trained over 34,000 individuals and distributed over 26,000 naloxone kits, with 2600 reported overdose reversals since 2004 [21]. Despite policies to increase the availability of naloxone and encourage PWID to call for help in response to overdose, it is not known if these policies are effective in real-world settings.

While Tobin et al. [18] characterized lifetime experience with the naloxone cascade, potential overdose responders need continuous and consistent access to naloxone in order to effectively implement this life-saving intervention. To examine continuous and consistent access to naloxone in PWID, we characterized recent experience with naloxone, including receipt of training and naloxone supply, use of naloxone after a witnessed overdose, and regular possession of naloxone among PWID in Baltimore, Maryland. The goal of the analysis was to identify factors associated with gaps in naloxone implementation to suggest opportunities to expand access to and use of naloxone among those most likely to respond to an overdose.

## Methods

### Study population

The individuals included in this study are participants of the ALIVE (AIDS Linked to the IntraVenous Experience) Study, a community-based cohort of PWID in Baltimore ongoing since 1988, described in detail elsewhere [22]. Briefly, the study enrolled n = 2,398 in 1988–89, n = 434 in 1994–95, n = 295 in 1998, n = 1,009 in 2005–08 and n = 830 in 2015–18. Inclusion criteria for participation included being at least 18 years of age and reporting a history of injection drug use in the past 1 to 10 years (criteria has changed over past 30 years of study to capture shifts in drug use patterns). Participants were recruited via community outreach at syringe services programs, community health fairs, drug treatment programs, community health and HIV clinics, and other community events. The current analysis was restricted to 915 participants who completed a study visit between January-June 2018 when a survey on overdose prevention strategies was administered. This study was approved by the Johns Hopkins Bloomberg School of Public Health Institutional Review Board and all participants provided written informed consent.

### Measures

As part of their participation in the ALIVE study, individuals complete bi-annual follow-up visits with standardized survey assessments, either interviewer-administered or collected via audio computer-assisted self-interview (ACASI). Assessments administered during baseline study visits collect information regarding socio-demographic characteristics (e.g., age, sex, race, marital status, educational attainment). During follow-up visits, participants complete additional assessments with respect to the prior six months regarding socio-demographic characteristics (e.g., residential location, income, employment, homelessness), substance use (opioids including heroin or non-medical prescription opioids, cocaine, marijuana) and the modes of administration of substances used (injection, snorting, smoking), alcohol and tobacco use, alcohol use disorders (using the Alcohol Use Disorders Identification Test), experience of non-fatal overdose, depressive symptoms (using the Center for Epidemiologic Studies Depression Scale), use of syringe services provision/needle exchange, alcohol or drug treatment (including inpatient detox, outpatient treatment, recovery groups, any alcohol dependence treatment and/or methadone), emergency room visits, outpatient visits, opioid agonist therapy (OAT; methadone and buprenorphine treatment), and whether the participant has a regular primary care provider. HIV testing is done semi-annually among all HIV-negative participants.

For this study, a brief survey designed to assess exposure to naloxone training, access and use was administered to participants in active follow-up in ALIVE starting in August 2017. Questions assessed whether participants had received any information or training about naloxone in the prior six months, the source of that training and whether anyone they knew had also received training. Additional questions asked participants whether they had received a supply of naloxone, usually carried a supply of naloxone, the source of the naloxone, whether or not they had administered naloxone to anyone in the prior six months, what happened immediately following the administration of naloxone, including whether anyone called 911 for emergency assistance, and whether they had received naloxone after an overdose. Finally, participants were asked whether they were aware of a law that protects them from criminal charges should they call for help following an overdose (e.g., the Good Samaritan law) and whether it is safe to give someone naloxone if they do not need it (“Do you think you could hurt someone if you gave them Narcan when they did not need it?”). (Full questionnaire is attached in supplemental materials).

### Statistical analysis

Descriptive statistics were used to characterize the sample population. We also used descriptive statistics to characterize experience with overdose prevention in terms of receipt of: 1) training: learned how to use naloxone in the prior six months; 2) access: receipt of supply of, or prescription for naloxone (of any form), in the past six months; 3) administration: used naloxone in the prior six months to respond to a witnessed overdose; and 4) possession: usually carried a supply of naloxone in the prior six months. We examined the proportion recently experiencing these outcomes overall and separately among those actively using any substance by any route of administration (e.g., heroin, cocaine, crack, speedball, non-medical prescription drugs). Similarly, we characterized calling 911 following the use of naloxone, knowledge of the Good Samaritan law, knowledge regarding potential harm from naloxone exposure, and self-reported experiences of overdose and receipt of naloxone. Importantly, each outcome was considered independent rather than as a subset of the prior meaning. For example, as supply could have been received more than 6 months ago and used within the last 6 months, we did not assume that using naloxone required having received a supply for the analysis.

We estimated unadjusted odds ratios using logistic regression models to identify the socio-demographic, substance use and healthcare utilization factors associated with each of the four separate outcomes described above: training, access, administration, and possession. Adjusted analysis sought to determine whether those who reported experiencing a non-fatal overdose in the prior six months also reported naloxone training, access, administration, or possession in the prior six months. Therefore, adjusted models included factors known to be associated with overdose among PWID populations *a priori* including demographic factors (e.g., age, sex, race), substance use, and utilization of services. Due to the limited number of participants who had administered naloxone to someone else in the prior 6 months (n = 85), we used Chi-square and Wilcoxon rank sum tests to identify factors associated with calling 911 when giving naloxone.

## Results

### Sample characteristics

A total of 915 participants, whose characteristics are displayed in Table 1, completed the survey regarding naloxone. The median age of participants was 56 years, 34% were female, 85% were African American, and approximately half (47%) had a high school diploma. In terms of substance use, while nearly half (47%) reported any alcohol use in the prior six months, 17% had a score of 8 or higher on the AUDIT, suggesting harmful alcohol use or dependence. Thirty-one percent were actively injecting drugs in the six months prior to the survey, with 153 (17%) injecting less than daily and 150 (14%) injecting daily or more frequently. Forty-one percent (n = 376) reported any cocaine use, 38% (n = 348) reported any opioid use, and 24% (n = 223) reported both alcohol and opioid use. The vast majority of the sample (91%, n = 835) reported that they had a regular primary care provider, 77% reported an outpatient health care visit in the prior six months, and almost half (47%) reported any alcohol or drug treatment.

**Table 1:** Socio-demographic characteristics, substance use, and health care utilization and engagement among n = 915 PWID in Baltimore, Maryland.

	N (%)
Socio-demographic characteristics (time-fixed)	
Median age, in years	56 (50–61)
Female (vs. Male)	310 (34)
Non-urban residence (vs. Baltimore City)	109 (12)
African American (vs. other race)	775 (85)
Ever married (vs. never)	412 (47)
High school education (vs. less than high school)	427 (47)
HIV-positive (vs. HIV-negative)	281 (31)
Socio-demographic characteristics (prior 6 months)	
Employed (vs. unemployed)	138 (15)
Income<$5K (vs. ≥$5K)	633 (70)
Homeless (vs. not)	74 (8)
Incarcerated (vs. not)	17 (2)
Depressive symptoms (CESD≥23)	253 (28)
Substance use (prior 6 months)	
Any cigarette use (vs. none)	684 (76)
Any alcohol use (vs. none)	427 (47)
AUDIT > = 8 (vs. <8)	157 (17)
Any injection (vs. none)	283 (31)
Frequency of injection (vs. none)	
Less than daily injection	153 (17)
Daily or more injection	150 (14)
Any cocaine use (vs. none)	376 (41)
Any opioid use (vs. none)	348 (38)
Marijuana use (vs. none)	154 (17)
Both opioid and alcohol use (vs. none)	223 (24)
Health care utilization and engagement (past 6 months)		
Attended syringe services provider (vs. did not attend)	141 (16)
Any alcohol or drug treatment (vs. none)	424 (47)
Has a regular primary care provider (vs. does not)	835 (91)
Emergency room visit (vs. none)	228 (25)
Inpatient hospitalization (vs. no)	125 (14)
Outpatient visit (vs. no)	701 (77)
Methadone treatment (vs. none)	379 (41)
Buprenorphine treatment (vs. none)	117 (13)

### Experiences with naloxone

In the prior six months, 46% of the sample (n = 421) reported having received training on naloxone, 38% (n = 346) reported access (i.e., having gotten a supply or a prescription for naloxone), 9% (n = 85) reported having administered naloxone to someone who had overdosed, and 9% (n = 82) reported possession, or usually carrying a supply of naloxone. Experiences with each naloxone outcome were more likely among the 50% of the sample reporting active substance use (n = 454): 56% received training, 47% reported access, 13% reported use after witnessing an overdose, and 12% usually carried a supply of naloxone.

### Training on naloxone

Of the 421 (46%) who reported having received training on naloxone, participants reported most often receiving training at drug treatment (n = 128, 30%), syringe services providers (n = 65, 16%), or by the health department (n = 72, 17%). Participants reported learning how to administer naloxone (n = 409, 97%), how to respond to an overdose (n = 392, 94%), how to perform rescue breathing (n = 258, 61%) and how to prevent an overdose (n = 253, 61%). Among the entire sample, 40% reported knowing someone else who also received training on naloxone. Factors positively associated with having received training in unadjusted analysis were high school education or greater, annual income less than $5,000, homelessness, depressive symptoms, active substance use (including injection drug use, any cocaine use, and any opioid use), recent overdose, and accessing substance use treatment and medical services (including use of syringe services provider, having had any alcohol or drug treatment, methadone and buprenorphine treatment, recent emergency room visit, and recent inpatient hospitalization) (Table 2). Older age, African American race and having ever been married were associated with decreased likelihood to of receiving naloxone training in past 6 months. Participants using both alcohol and opioids and those using only opioids were more likely to receive naloxone training in past 6 months compared to those only using alcohol or using neither substance (p<0.001).

**Table 2:** Unadjusted correlates (odds ratios and 95% confidence intervals) of naloxone implementation (training, access, administration, possession) in the prior six months among n = 915 PWID in Baltimore, Maryland.

	Training OR (95% CI)	Access OR (95% CI)	Administration OR (95% CI)	Possession OR (95% CI)
Socio-demographic characteristics (time-fixed)				
Age (per 5 yrs)	**0.77 (0.71, 0.83)**	**0.76 (0.71, 0.83)**	**0.83 (0.74, 0.93)**	**0.86 (0.76, 0.96)**
Female (vs. Male)	1.23 (0.93, 1.61)	1.08 (0.81, 1.43)	**1.66 (1.06, 2.61)**	1.43 (0.90, 2.27)
Non-urban residence (vs. Baltimore City)	0.92 (0.61, 1.37)	0.95 (0.63, 1.44)	0.87 (0.42, 1.80)	0.79 (0.37, 1.69)
African American (vs. other race)	**0.41 (0.29, 0.60)**	**0.39 (0.27, 0.57)**	**0.55 (0.32, 0.94)**	0.61 (0.35, 1.07)
Ever married (vs. never)	**0.66 (0.51, 0.87)**	**0.75 (0.57, 0.99)**	**0.59 (0.36, 0.96)**	0.72 (0.28, 1.90)
High school education (vs. less than high school)	**1.29 (1.00, 1.68)**	1.18 (0.90, 1.54)	0.92 (0.58, 1.43)	1.15 (0.73, 1.82)
HIV-positive (vs. HIV-negative)	0.84 (0.63, 1.12)	0.96 (0.72, 1.28)	0.82 (0.50, 1.36)	**0.56 (0.32, 0.98)**
Socio-demographic characteristics (prior 6 months)				
Employed (vs. unemployed)	0.83 (0.57, 1.19)	0.83 (0.57, 1.22)	0.92 (0.48, 1.74)	0.86 (0.44, 1.67)
Low income (<$5K vs. ≥$5K)	**1.40 (1.04, 1.86)**	1.39 (1.03, 1.88)	1.32 (0.79, 2.21)	1.25 (0.75, 2.11)
Homeless (vs. not)	**2.64 (1.59, 4.37)**	**2.46 (1.52, 3.98)**	**2.04 (1.05, 3.95)**	1.26 (0.58, 2.72)
Incarcerated (vs. not)	1.69 (0.64, 4.49)	1.47 (0.56, 3.85)	1.31 (0.29, 5.82)	—
Depressive symptoms (CESD≥23 vs. CESD<23)	**1.51 (1.12, 2.03)**	**1.42 (1.06, 1.91)**	1.15 (0.70, 1.89)	0.98 (0.39, 2.48)
Substance Use (prior 6 months)				
Any cigarette use (vs. none)	1.34 (0.99, 1.83)	**1.64 (1.18, 2.28)**	1.38 (0.78, 2.43)	1.75 (0.61, 5.08)
Any alcohol use (vs. none)	0.91 (0.70, 1.19)	0.92 (0.70, 1.20)	1.27 (0.81, 2.00)	0.94 (0.38, 2.31)
AUDIT > = 8 (vs. <8)	0.94 (0.66, 1.32)	0.96 (0.67, 1.36)	1.12 (0.63, 1.99)	0.97 (0.43, 2.19)
Any injection (vs. none)	**2.31 (1.73, 3.08)**	**2.37 (1.78, 3.16)**	**2.76 (1.76, 4.34)**	1.18 (0.45, 3.07)
Frequency of injection (vs. none)				
Less than daily injection	**1.78 (1.23, 2.53)**	**1.80 (1.26, 2.59)**	1.59 (0.85, 2.96)	1.09 (0.58, 2.06)
Daily or more injection	**3.22 (2.16, 4.81)**	**3.29 (2.23, 4.86)**	**4.38 (2.61, 7.36)**	**2.13 (1.22, 3.73)**
Any cocaine use (vs. none)	**2.35 (1.80, 3.08)**	**2.23 (1.70, 2.95)**	**2.30 (1.46, 3.63)**	**1.92 (1.22, 3.04)**
Any opioid use (vs. none)	**2.54 (1.93, 3.35)**	**2.71 (2.05, 3.58)**	**2.81 (1.77, 4.44)**	**2.32 (1.48, 3.71)**
Marijuana use (vs. none)	1.38 (0.98, 1.95)	1.17 (0.82, 1.67)	**1.83 (1.09, 3.07)**	1.43 (0.82, 2.48)
Overdose (vs. none)	**2.46 (1.33, 4.55)**	**2.41 (1.34, 4.36)**	**3.59 (1.79, 7.20)**	1.18 (0.45, 3.07)
Health care utilization and engagement (past 6 months)						
Attended syringe services provider (vs. did not attend)	**2.76 (1.89, 4.03)**	**3.11 (1.15, 4.50)**	**4.20 (2.59, 6.79)**	**1.87 (1.09, 3.22)**
Alcohol or drug treatment (vs. none)	**2.81 (2.14, 3.67)**	**2.79 (2.12, 3.68)**	**2.06 (1.29, 3.27)**	**2.01 (1.26, 3.21)**
Has a regular primary care provider (vs. does not)	0.67 (0.42, 1.07)	0.68 (0.43, 1.09)	0.79 (0.38, 1.64)	0.76 (0.36, 1.57)
Emergency room visit (vs. none)	**2.03 (1.49, 2.74)**	**1.77 (1.31, 2.40)**	**1.84 (1.15, 2.94)**	**2.07 (1.29, 3.32)**
Inpatient hospitalization (vs. no)	**2.09 (1.42, 3.08)**	**1.52 (1.04, 2.22)**	**1.81 (1.03, 3.16)**	**2.78 (1.64, 4.69)**
Outpatient visit (vs. no)	0.91 (0.67, 1.24)	1.16 (0.84, 1.60)	1.43 (0.80, 2.55)	1.13 (0.65, 1.97)
Methadone treatment (vs. none)	**2.70 (2.06, 3.54)**	**2.53 (1.92, 3.34)**	**1.95 (1.24, 3.06)**	**1.91 (1.21, 3.03)**
Buprenorphine treatment (vs. none)	**1.55 (1.05, 2.29)**	**1.48 (1.00, 2.19)**	**2.33 (1.35, 4.01)**	**1.92 (1.08, 3.40)**

### Accessing naloxone

Of the 346 (38%) who received a supply or prescription of naloxone in the prior 6 months, most reported access from a drug treatment program (n = 83, 24%), the health department (n = 68, 20%), a syringe services program (n = 65, 19%), a doctor’s office (n = 30, 9%), a pharmacy without a prescription (n = 14, 4%), or another unspecified source (n = 67, 19%). Seventy-seven percent (n = 324) of the 421 who received naloxone training in past 6 months also reported having received supply of naloxone in past 6 months, compared to only 4% (n = 22) of the 493 who had not received training (p<0.001). Similarly, 93% of the 346 who received naloxone also reported training, while only 7% reported a supply of naloxone without training. Factors positively associated with access to naloxone in the prior six months in unadjusted analysis were homelessness, depressive symptoms, active substance use (including cigarette use, injection drug use, any opioid use, any cocaine use), recent overdose, and accessing medical services (including use of syringe services provider, any recent alcohol or drug treatment (including buprenorphine and methadone), recent emergency room visit and recent hospitalization) (Table 2). Older and African-American participants and those who were ever married were less likely to receive supply of naloxone in unadjusted analysis (Table 2).

### Administering naloxone

Of the 85 (9%) who reported administering naloxone to someone else in the prior 6 months, half (n = 43, 51%) reported having given naloxone to only one person, while 31% (n = 26) reported having given naloxone to two people, and the remainder (n = 16, 18%) reported having given naloxone to three or more people in the prior six months. 76 (89%) had received naloxone training in past 6 months. Most reported that the person to whom they administered naloxone “woke up” (n = 68, 80%). Other responses included: “nothing happened” (n = 14, 16%), “the person passed out again” or “they administered more naloxone” (n = 14, 16%), “they passed out, but the person did not have any additional naloxone” (n = 2, 2%), “the person had a bad reaction” (n = 3, 4%), or the individual died at the scene (n = 3, 4%). Of the 85 participants who reported having administered naloxone in the prior six months, 56 (66%) reported that they called 911 after the administration. None of the above responses about what happened following naloxone administration was associated with calling 911. Compared to those who did not call 911 after administering naloxone, those who did were less likely to report cocaine use (p = 0.03), opioid use (p = 0.05), or having attended a syringe services provider (p = 0.03).

Factors positively associated with administering naloxone in the prior six months in unadjusted analysis included female sex, homelessness, active substance use (injection drug use, any cocaine use, any opioid use, marijuana use), and having had an overdose (Table 2). Also positively associated were use of syringe services program, having received any alcohol or drug treatment, having had an emergency room visit, having had an inpatient hospitalization, having received methadone or buprenorphine treatment in the prior six months. Older age, African American race and ever being married were negatively associated with having given naloxone in the past 6 months.

### Naloxone possession

Although only 82 (9% of total sample) participants reported regularly carrying naloxone, of the 346 who reported receiving a supply in the prior 6 months, 18% (n = 62) reported possession. However, of the 85 who reported administering naloxone to someone in the last 6 months, only 19% reported that they regularly carried it. Daily or more frequent injection drug use, cocaine use, opioid use, use of syringe services provider, having had any alcohol or drug treatment, a recent emergency room visit, recent inpatient hospitalization, and methadone and buprenorphine treatment were positively associated with regularly carrying naloxone (Table 2). Older age and HIV-positive status were negatively associated with carrying a supply of naloxone.

### Adjusted results

Table 3 presents adjusted odds ratios of naloxone implementation (training, access, administration, and possession) in the prior six months. Older age was negatively associated with having received training and a supply of naloxone. Alcohol and drug treatment was positively associated with all four naloxone outcomes in adjusted analysis. High school education (aOR = 1.37, 95% CI: 1.01, 1.85) and a recent emergency room visit (aOR = 1.57, 95% CI: 1.03, 2.38) were associated with having received training on naloxone. Those reporting recent marijuana use (aOR = 1.91, 95% CI: 1.01, 3.60), and those who reported use of syringe services (aOR = 2.71, 95% CI: 1.22, 6.02) were more likely to have reported having administered naloxone. Recent opioid use (aOR = 2.10, 95% CI: 1.03, 4.28) and recent inpatient hospitalization were associated with regularly carrying naloxone (aOR = 2.68, 95% CI: 1.26, 5.67).

**Table 3:** Adjusted^a^ odds ratios (aOR) and 95% confidence intervals (CI) of correlates of naloxone implementation (training, access, administration, and possession) in the prior six months among n = 915 PWID in Baltimore, Maryland.

	TrainingaOR (95% CI)	Access aOR (95% CI)	Administration OR (95% CI)	PossessionaOR (95% CI)
Time-fixed characteristics				
Age (per 5 yrs)	**0.87 (0.78, 0.97)**	**0.86 (0.77, 0.96)**	0.93 (0.79, 1.09)	0.90 (0.77, 1.07)
Female (vs. male)	1.15 (0.83, 1.59)	0.97 (0.70, 1.36)	1.48 (0.86, 2.52)	1.24 (0.72, 2.11)
African American (vs. other)	0.92 (0.56, 1.51)	0.78 (0.48, 1.28)	1.14 (0.52, 2.49)	1.26 (0.57, 2.80)
Ever married (vs. never)	0.84 (0.62, 1.14)	0.97 (0.71, 1.33)	0.68 (0.40, 1.16)	0.76 (0.45, 1.27)
High school education (vs. less than high school)	**1.37 (1.01, 1.85)**	1.21 (0.88, 1.65)	0.97 (0.58, 1.61)	1.19 90.71, 1.97)
HIV-positive (vs. HIV-negative)	0.95 (0.69, 1.32)	1.23 (0.88, 1.72)	1.11 (0.63, 1.95)	0.64 (0.35, 1.17)
Recent characteristics (past 6 months)				
Overdose (vs. none)	0.95 (0.47, 1.96)	0.99 (0.50, 1.97)	1.35 (0.56, 3.23)	0.68 (0.23, 2.00)
Low-income (<$5K vs. ≥$5K)	0.95 (0.68, 1.33)	0.94 (0.66, 1.33)	0.82 (0.46, 1.47)	0.99 (0.56, 1.77)
Homeless (vs. not)	1.62 (0.89, 2.94)	1.27 (0.71, 2.23)	1.08 (0.47, 2.46)	0.38 (0.12, 1.17)
Depressive symptoms (CESD≥23 vs. CESD<23)	0.85 (0.60, 1.21)	0.83 (0.58, 1.19)	0.80 (0.45, 1.43)	0.79 (0.44, 1.41)
Frequency of injection (vs. none)				
Less than daily injection	1.02 (0.58, 1.80)	0.90 (0.51, 1.60)	0.63 (0.22, 1.78)	0.38 (0.14, 1.02)
Daily or more injection	1.68 (0.88, 3.22)	1.54 (0.81, 2.92)	2.06 (0.75, 5.67)	0.83 (0.31, 2.23)
Any cocaine use (vs. none)	1.28 (0.87, 1.88)	1.17 (0.79, 1.73)	0.80 (0.42, 1.53)	0.97 (0.52, 1.83)
Any opioid use (vs. none)	1.23 (0.79, 1.91)	1.38 (0.87, 2.16)	1.03 (0.47, 2.27)	**2.10 (1.03, 4.28)**
Marijuana use (vs. none)	0.95 (0.62, 1.46)	0.82 (0.53, 1.26)	**1.91 (1.01, 3.60)**	1.49 (0.78, 2.83)
Attended syringe services provider (vs. did not attend)	0.99 (0.57, 1.72)	1.28 (0.74, 2.20)	**2.71 (1.22, 6.02)**	1.38 (0.57, 3.32)
Any alcohol or drug treatment (vs. none)^b^	**2.69 (1.96, 3.69)**	**2.77 (1.99, 3.86)**	**2.99 (1.63, 5.50)**	**2.01 (1.13, 3.56)**
Emergency room visit (vs. none)	**1.57 (1.04, 2.38)**	1.50 (0.98, 2.28)	1.45 (0.76, 2.74)	1.14 (0.57, 2.27)
Inpatient hospitalization (vs. no)	1.59 (0.94, 2.68)	1.15 (0.69, 1.93)	1.22 (0.57, 2.59)	**2.68 (1.26, 5.67)**

^a^ Models were adjusted for all variables in the table.

^b^ Includes any alcohol or drug treatment, including methadone prescription.

### Overdose experiences

Among the 3% (n = 29) of participants who reported experiencing a non-fatal overdose in past six months, 82% (n = 23) reported having been given naloxone at the time of the overdose. Among these, 69% (n = 20) reported that an ambulance responded and 48% (n = 14) went to the emergency room. Naloxone was given for all of the overdoses when no one called for help, compared to only 70% of the overdoses when someone called for help (p = 0.04). While 59% (n = 17) had received supply of naloxone in past six months, only 28% (n = 8) were carrying a supply of naloxone at time of their last overdose. 28% (n = 8) of participants who reported a recent overdose were given a supply of or prescription for naloxone afterwards.

### Knowledge and attitudes about naloxone

A total of 59% of participants (n = 541) reported that they were aware of the law that would protect them if they called for help following an overdose (i.e., Good Samaritan laws). Approximately half of the sample (n = 525, 51%) believed that administering naloxone if not needed could be harmful to the recipient, which did not differ by whether participants reported having received training.

## Discussion

In a community-based cohort of current and former PWID living in Baltimore, nearly half reported recently receiving naloxone training and many had recently received a supply of naloxone (38%), but recent use (9%) and regular possession (9%) of naloxone were much less frequently reported. These findings support prior research on lifetime engagement in the ‘naloxone cascade’ in Baltimore [18] and other settings [23], where awareness of naloxone was high (70–90%), but possession and use were low (20–30%). Unfortunately, the pervasiveness of fentanyl in the Baltimore drug supply [24] and the resulting rise in overdose mortality rates locally demonstrate that there is a dire need for not only OEND, but ready and consistent access to naloxone.

There are several potential explanations for why few PWID in our study reported regularly carrying naloxone. First, trainings do not always provide a supply of naloxone. Second, PWID may fear, perceive or experience stigma while accessing naloxone at pharmacies [25] or while carrying naloxone. Finally, PWID may need access to a more consistent supply of naloxone, as prior research demonstrated that 18% of those on medications for opioid use disorder in New Mexico who had received training and a supply of naloxone had already used their naloxone within 6 months of training [26]. Notably, attendance at SSPs was not associated with self-reported receipt of naloxone training, yet these individuals were more likely to have given naloxone and previous work has shown that provision of naloxone at SSPs is acceptable to PWID [27]. This may reflect receipt of informal education via peer networks.

This study builds on previous work by examining associations between regularly carrying naloxone and substance use and utilization of substance use treatment and other health services. Those reporting active opioid use and recent inpatient hospitalization were more likely to report regularly carrying naloxone. Those accessing any kind of treatment for substance use disorder were more likely to report all naloxone outcomes, including regular carrying. Taken together, these results suggest that implementation of naloxone among people currently injecting drugs, those with severe health conditions (requiring hospitalization) and those accessing addiction treatment settings has been successful. Further work is needed to identify additional settings for OEND that encourage carrying and effectively using naloxone among persons most at risk for experiencing overdose. Even brief (5–10 minute) OEND trainings may be both effective and practical [28], in several points of care, such as syringe services programs and other healthcare settings.

That PWID reporting recent emergency department (ED) encounters were more likely to have received information, but no more likely to report a supply or to carry naloxone may reflect missed opportunities to provide naloxone to those at highest risk. Similarly, the lack of an association between recent non-fatal overdose and carrying naloxone may suggest missed opportunities for intervention following overdose, given that nearly half of overdose events in this study resulted in an ED visit and only 28% of participants who reported overdose were given a supply of or prescription for naloxone after experiencing a nonfatal overdose. Trainings by first responders or in ED could be particularly effective given that a history of overdose is a strong predictor of subsequent overdose, though research suggests that witnessing an overdose is a stronger predictor of naloxone uptake than personal experience of overdose [23,29–31]. Prior research has also demonstrated that patients generally accept take-home naloxone kits when offered in the ED [29], however there is some evidence to show that overdose prevention interventions among those at risk in the ED may not have an impact on subsequent overdose [32]. Efforts to promote initiation of opioid agonist treatment in the ED may be an efficient and effective strategy to reduce overdose mortality [33]. Additional research to identify and implement effective strategies for overdose prevention among PWID in the ED is necessary.

Finally, despite knowledge of Maryland’s Good Samaritan law, only half of participants in this study called 911 after giving naloxone for overdose, with lower 911 calling among those using cocaine, opioids and more frequent injection drug use. While Good Samaritan laws have been enacted in 34 states [34], PWID may remain hesitant to call 911 after responding to an overdose due to fear of repercussions of interacting with EMS, including fear of arrest and incarceration and a general distrust of police and the legal system [35]. Furthermore, nearly half of the participants in this study believed that administering naloxone if it were not needed could cause harm, pointing to ongoing gaps in knowledge. Although half of the respondents in this study reported having received information or training about naloxone, further work is needed to correct misperceptions and to encourage those responding to overdose to call 911. Additional efforts may be indicated to train first responders and police in the importance of protections for those reporting overdose.

Of note, this study sample was predominantly African American, a group in which overdose mortality is rising in Baltimore City [36] as well as nationally [37]. African Americans were significantly less likely to receive training, access or give naloxone in univariable analysis, although this association did not hold in multivariable analysis. Further work is needed to explore whether African-American participants are less likely to access or use naloxone and if so, the mechanisms explaining decreased access and use. High rates of incarceration among African-Americans in Baltimore City [38] and nationally [39] (although low among this sample at this point in time) may lead to fear of police involvement and incarceration from carrying or giving naloxone and from calling 911 among African American PWID [35,40].

This study has several limitations. The study sample consisted of current and former PWID in a single urban setting and may not be generalizable to other populations of people who use or inject drugs. Data were self-reported and may be biased due to recall or social desirability. Our estimates of the proportion of PWID who have received training or supply of naloxone is likely an underestimate, as it did not capture those who received training or supply of naloxone more than six months previously. Additionally, our survey did not differentiate between types of naloxone training and was not designed to assess if participants who received training were prepared to use naloxone. We cannot distinguish the reasons why PWID did not carry naloxone, whether it was due to lack of access or stigma, for example. Finally, we did not assess whether participants witnessed an overdose, and our assessment of self-reported overdose did not include whether training was offered to the participant following the overdose. Thus, we cannot restrict our analysis of correlates of administering naloxone to only those who witnessed an overdose and similarly, we are limited to examining 911 calling only among participants who reported that they had administered naloxone.

## Conclusions

Current and former PWID reporting recent opioid use were more likely to report that they regularly carry naloxone. However, those experiencing a recent non-fatal overdose were no more likely than those not reporting an overdose to have received overdose prevention education, a supply of naloxone, recent use of naloxone or regular possession. Further work is needed to encourage PWID to carry and effectively use naloxone to decrease rates of fatal opioid overdose. While engagement in treatment for substance or alcohol use disorder or OAT was associated with all naloxone outcomes in this study, first responders to overdose, the ED and SSP may be settings in which access to naloxone could be expanded to improve implementation and overdose prevention among PWID.

## Supporting information

S1 File The ALIVE study follow-up overdose questionnaire. (DOCX)
