# Supplementary material for: Fatal overdose prevention and experience with naloxone: A cross-sectional study from a community-based cohort of people who inject drugs in Baltimore, Maryland
Source: PLoS One. 2020 Mar 11;15(3):e0230127. doi: 10.1371/journal.pone.0230127 (PMC7065783; doi:10.1371/journal.pone.0230127)
Supplement: S1 File — (DOCX) [file pone.0230127.s001.docx]

THE ALIVE STUDY

FOLLOW-UP OVERDOSE QUESTIONNAIRE

SUBJECT ID: ____ ____ ____ ____ ____ 1

ALIVE STUDY VISIT: ____ ____ ____ 6

DATE OF INTERVIEW: ____ ____ / ____ ____ / ____ ____ 9

INTERVIEWER’S INITIALS: ____ ____ ____ 15

START TIME: ____ ____ : ____ ____ 18

**Read to the Participant:**

I would now like to ask you some questions about Narcan. Narcan is a drug that reverses overdose from heroin and other opiate drugs. It is also called naloxone.

1. In the last six months, have you received information or training about Narcan?

| No | 0 (4) | 23 |
| --- | --- | --- |
| Yes | 1 |  |
| Don’t know | 7 (4) |  |
| Refused | 8 (4) |  |

2. Where did you receive information or training about Narcan?

**(Choose all that apply)**

| **INTERVIEWER READ:** | | No | Yes | Don’t know | Refused |  |
| --- | --- | --- | --- | --- | --- | --- |
| a. | Staying Alive program | 0 | 1 | 7 | 8 | 24 |
| b. | Needle exchange program | 0 | 1 | 7 | 8 | 25 |
| c. | Other community naloxone program | 0 | 1 | 7 | 8 | 26 |
| d. | Doctor’s office | 0 | 1 | 7 | 8 | 27 |
| e. | Drug treatment program | 0 | 1 | 7 | 8 | 28 |
| f. | Pharmacy | 0 | 1 | 7 | 8 | 29 |
| g. | Health department | 0 | 1 | 7 | 8 | 30 |
| h. | On the street | 0 | 1 | 7 | 8 | 31 |
| i. | Family member or a friend | 0 | 1 | 7 | 8 | 32 |
| h. | Other (specify):  ________________________ | 0 | 1 | 7 | 8 | 33 |

3. In the last six months, what information or training did you get about Narcan?

**(Choose all that apply)**

|  | **INTERVIEWER READ:** | No | Yes | Don’t know | Refused |  |
| --- | --- | --- | --- | --- | --- | --- |
| a. | How to decrease risk of overdose | 0 | 1 | 7 | 8 | 53 |
| b. | How to respond to an overdose | 0 | 1 | 7 | 8 | 54 |
| c. | How to do rescue breathing | 0 | 1 | 7 | 8 | 55 |
| d. | How to give Narcan (naloxone) | 0 | 1 | 7 | 8 | 56 |
| e. | Other (specify):  __________________________ | 0 | 1 | 7 | 8 | 57 |

4. In the last six months, has anyone you know received information or training about Narcan?

| No | 0 (8) |  | 77 |
| --- | --- | --- | --- |
| Yes | 1 |  |  |
| Don’t know | 7 (8) |  |  |
| Refused | 8 (8) |  |  |

5. Who do you know that has received information or training about Narcan?

**(Choose all that apply)**

| **INTERVIEWER READ:** | | No | Yes | Don’t know | Refused |  |
| --- | --- | --- | --- | --- | --- | --- |
| a. | Family member | 0 | 1 | 7 | 8 | 78 |
| b. | Friend | 0 | 1 | 7 | 8 | 79 |
| c. | Person you use drugs with | 0 | 1 | 7 | 8 | 80 |
| d. | Neighbor | 0 | 1 | 7 | 8 | 81 |
| e. | Other (specify):  ________________________ | 0 | 1 | 7 | 8 | 82 |

6. Where have people you know received information or training about Narcan?

**(Choose all that apply)**

| **INTERVIEWER READ:** | | No | Yes | Don’t know | Refused |  |
| --- | --- | --- | --- | --- | --- | --- |
| a. | Staying Alive program | 0 | 1 | 7 | 8 | 102 |
| b. | Needle exchange program | 0 | 1 | 7 | 8 | 103 |
| c. | Other community naloxone program | 0 | 1 | 7 | 8 | 104 |
| d. | Doctor’s office | 0 | 1 | 7 | 8 | 105 |
| e. | Drug treatment program | 0 | 1 | 7 | 8 | 106 |
| f. | Pharmacy | 0 | 1 | 7 | 8 | 107 |
| g. | Health department | 0 | 1 | 7 | 8 | 108 |
| h. | On the street | 0 | 1 | 7 | 8 | 109 |
| i. | Family member or a friend | 0 | 1 | 7 | 8 | 110 |
| h. | Other (specify):  ________________________ | 0 | 1 | 7 | 8 | 111 |

7. In the last six months, what information or training have people you know gotten

about Narcan?

**(Choose all that apply)**

|  | **INTERVIEWER READ:** | No | Yes | Don’t know | Refused |  |
| --- | --- | --- | --- | --- | --- | --- |
| a. | How to decrease risk of overdose | 0 | 1 | 7 | 8 | 131 |
| b. | How to respond to an overdose | 0 | 1 | 7 | 8 | 132 |
| c. | How to do rescue breathing | 0 | 1 | 7 | 8 | 133 |
| d. | How to give Narcan (naloxone) | 0 | 1 | 7 | 8 | 134 |
| e. | Other (specify):  ____________________ | 0 | 1 | 7 | 8 | 135 |

8. In the last six months, did you get a supply of or prescription for Narcan?

| No | 0 (10 |  | 155 |
| --- | --- | --- | --- |
| Yes | 1 |  |  |
| Don’t know | 7 (10) |  |  |
| Refused | 8 (10) |  |  |

9. Where did you get the Narcan from? **(Choose all that apply)**

| **INTERVIEWER READ:** | | No | Yes | Don’t know | Refused |  |
| --- | --- | --- | --- | --- | --- | --- |
| a. | Staying Alive program | 0 | 1 | 7 | 8 | 156 |
| b. | Needle exchange program | 0 | 1 | 7 | 8 | 157 |
| c. | Other community naloxone program | 0 | 1 | 7 | 8 | 158 |
| d. | Prescription from doctor’s office | 0 | 1 | 7 | 8 | 159 |
| e. | Drug treatment program | 0 | 1 | 7 | 8 | 160 |
| f. | Directly from pharmacy (no prescription) | 0 | 1 | 7 | 8 | 161 |
| g. | Health department | 0 | 1 | 7 | 8 | 162 |
| h. | Other (specify):  ________________________ | 0 | 1 | 7 | 8 | 163 |

10. Are you currently carrying a supply of Narcan?

| No | 0 |  | 278 |
| --- | --- | --- | --- |
| Yes | 1 |  |  |
| Don’t know | 7 |  |  |
| Refused | 8 |  |  |

11. In the last six months, did you give Narcan to a person who overdosed?

| No | 0 (15) |  | 183 |
| --- | --- | --- | --- |
| Yes | 1 |  |  |
| Don’t know | 7 (15) |  |  |
| Refused | 8 (15) |  |  |
|  |  |  |  |

12. In the last six months, how many different occasions did you give someone Narcan?

#____ ____ 184

97 Don’t know 98 Refused

13. In the last six months, did any of the following things happen immediately after the last time you gave a person Narcan? **(Choose all that apply)**

| **INTERVIEWER READ:** | | No | Yes | Don’t know | Refused |  |
| --- | --- | --- | --- | --- | --- | --- |
| a. | Person woke up | 0 | 1 | 7 | 8 | 186 |
| b. | Nothing | 0 | 1 | 7 | 8 | 187 |
| c. | Person passed out again so you gave them more Narcan | 0 | 1 | 7 | 8 | 188 |
| d. | Person passed out again, but you were out of Narcan | 0 | 1 | 7 | 8 | 189 |
| e. | Person had a bad reaction to the Narcan (e.g., vomiting, seizures, became violent) | 0 | 1 | 7 | 8 | 190 |
| f. | Person who got Narcan used drugs again that same day | 0 | 1 | 7 | 8 | 191 |
| g. | Person died at the scene | 0 | 1 | 7 | 8 | 192 |
| h. | Other (specify):  _______________________ | 0 | 1 | 7 | 8 | 193 |

14. Did you call 911 after you gave the person Narcan?

| No | 0 |  | 213 |
| --- | --- | --- | --- |
| Yes | 1 |  |  |
| Don’t know | 7 |  |  |
| Refused | 8 |  |  |

15. Are you aware of the law that protects you from criminal charges

if you call for help after someone has a drug overdose?

| No | 0 |  | 214 |
| --- | --- | --- | --- |
| Yes | 1 |  |  |
| Don’t know | 7 |  |  |
| Refused | 8 |  |  |

16. Do you think you could hurt someone if you gave them Narcan when they

did not need it?

| No | 0 |  | 215 |
| --- | --- | --- | --- |
| Yes | 1 |  |  |
| Don’t know | 7 |  |  |
| Refused | 8 |  |  |

**Overdose**

17. Have you used fentanyl from the street (by any route) in the last six months?

| No | 0 (24) | 216 |
| --- | --- | --- |
| Yes | 1 |  |
| Refused | 7 (24) |  |
| Don’t know | 8 (24) |  |

|  | Have you used fentanyl by the following route in the last six months? | |  |
| --- | --- | --- | --- |
| 18. Fentanyl alone by injection | 0 | 1 | 217 |
| 19. Fentanyl and heroin together by injection | 0 | 1 | 218 |
| 20. Fentanyl by snorting | 0 | 1 | 219 |
| 21. Fentanyl and heroin together by snorting | 0 | 1 | 220 |
| 22. Fentanyl taken orally | 0 | 1 | 221 |
| 23. Fentanyl patch not from prescription | 0 | 1 | 222 |

Read to the Participant:

**I am now going to ask you some questions are about your experience with drug overdose. When I say “overdose,” I mean a situation where, after using, you or another person passed out and couldn’t wake up. The lips of the person who overdosed might have turned blue and their breathing was very slow or stopped.**

24. Have you had a drug overdose in the last six months? 223

| No | 0 (END) |
| --- | --- |
| Yes | 1 |
| Refused | 7 (END) |
| Don’t know | 8 (END) |

25. The last time you overdosed, did …

**(Choose all that apply)**

| **INTERVIEWER READ:** | | No | Yes | Don’t know | Refused |  |
| --- | --- | --- | --- | --- | --- | --- |
| a. | no one called for help? | 0 | 1 | 7 | 8 | 224 |
| b. | an ambulance come? | 0 | 1 | 7 | 8 | 225 |
| c. | you go to the emergency room? | 0 | 1 | 7 | 8 | 226 |
| d. | did someone give you Narcan? | 0 | 1 | 7 | 8 | 227 |
| e. | Other (specify):  ______________ | 0 | 1 | 7 | 8 | 228 |

26. After your last overdose, did you receive a referral to a drug treatment program?

| No | 0 |  | 248 |
| --- | --- | --- | --- |
| Yes | 1 |  |  |
| Don’t know | 7 |  |  |
| Refused | 8 |  |  |

27. Did you seek drug treatment in the 30 days after your last overdose?

| No | 0 |  | 249 |
| --- | --- | --- | --- |
| Yes | 1 |  |  |
| Don’t know | 7 |  |  |
| Refused | 8 |  |  |

28. Did you start drug treatment after your last overdose?

250

| No | 0 |  |  |
| --- | --- | --- | --- |
| Yes | 1 |  |  |
| Don’t know | 7 |  |  |
| Refused | 8 |  |  |

29. Were you in a drug treatment program at the time of your last overdose?

| No | 0 (30) |  | 251 |
| --- | --- | --- | --- |
| Yes | 1 |  |  |
| Don’t know | 7 (30) |  |  |
| Refused | 8 (30) |  |  |

30. What type of drug treatment program were you in? **(Choose all that apply)**

| **INTERVIEWER READ:** | | No | Yes | Don’t know | Refused |  |
| --- | --- | --- | --- | --- | --- | --- |
| a. | Methadone program | 0 | 1 | 7 | 8 | 252 |
| b. | Suboxone (buprenorphine) program | 0 | 1 | 7 | 8 | 253 |
| c. | Inpatient drug treatment without medication | 0 | 1 | 7 | 8 | 254 |
| d. | Outpatient drug treatment without medication | 0 | 1 | 7 | 8 | 255 |
| e. | Other (specify):  _______________________ | 0 | 1 | 7 | 8 | 256 |

31. At the time of your last overdose, did you have a supply of or prescription for Narcan?

| No | 0 |  | 276 |
| --- | --- | --- | --- |
| Yes | 1 |  |  |
| Don’t know | 7 |  |  |
| Refused | 8 |  |  |

32. After your last overdose, were you given a prescription for or supply of Narcan?

| No | 0 | 277 |
| --- | --- | --- |
| Yes | 1 |  |
| Don’t know | 7 |  |
| Refused | 8 |  |

33. After your last overdose, how long was it before you started using drugs again?

| That same day | 1 |  | 279 |
| --- | --- | --- | --- |
| The next day | 2 |  |  |
| That week | 3 |  |  |
| 1-3 weeks later | 4 |  |  |
| 1 month later or more | 5 |  |  |
| Haven’t started using drugs again | 6 |  |  |
| Don’t know | 7 |  |  |
| Refused | 8 |  |  |

**END TIME:** ____ ______ : ______ ______ 280

VERSION 5.0 285
